# Silica Nanoparticles Elicit Pulmonary Inflammation via STING-Dependent Activation of NF-κB p65 Signaling Pathway

**DOI:** 10.3390/biom16091273

**Published:** 2026-09-03

**Authors:** Junyi Gao, Fei Wu, Bing Li, Yuhang Ji, Hongxu Dong, Dongfeng Wang, Zixuan Liu

**Affiliations:** 1 School of Life Science and Technology, Shandong Second Medical University, Weifang 261053, China; gaojunyi@stu.sdsmu.edu.cn (J.G.); wufei@sdsmu.edu.cn (F.W.); libing20230052@stu.sdsmu.edu.cn (B.L.); 20247440007ji@stu.sdsmu.edu.cn (Y.J.); dhx20250054@stu.sdsmu.edu.cn (H.D.); 2 Laboratory of Morphology, School of Basic Medical Science, Shandong Second Medical University, Weifang 261053, China; wangdongfeng@sdsmu.edu.cn

**Keywords:** SiNPs, STING, NF-κB, lung inflammation

## Abstract

Silica nanoparticles (SiNPs) are common nanoparticles that are widely used in industrial and medical applications. Inhalation exposure to SiNPs is frequently unavoidable in occupational and everyday settings. While the adverse effects of SiNPs on lung injury have been extensively documented, the intrinsic mechanisms underlying SiNPs-induced pulmonary inflammation remain incompletely understood. This study demonstrates that the stimulator of interferon genes (STING) plays an essential role in SiNPs-triggered lung inflammation. In vitro, SiNPs induced death of bone marrow-derived macrophages (BMDMs) and activated the STING pathway. Knockout of STING expression alleviated SiNPs-induced inflammatory responses and subsequent cell death. Further mechanistic investigations revealed that STING activation promotes nuclear translocation of NF-κB p65 and subsequent activation of the NF-κB pathway, ultimately driving the secretion of inflammatory cytokines. In vivo, SiNPs-induced NF-κB activation and inflammatory cell infiltration were significantly attenuated in STING-deficient (STING^−/−^) mice. These findings reveal that targeting the STING signaling pathway may represent a potential therapeutic strategy for mitigating lung inflammation caused by silica particles.

## 1. Introduction

Natural silica exists in both crystalline and amorphous forms, and ranks among the most abundant compounds on Earth. Occupational exposure to crystalline silica has been unequivocally linked to the development of silicosis [1]. Although regulatory measures and exposure control strategies have been implemented, new cases of silicosis continue to emerge, in part due to non-traditional exposure sources such as silica nanoparticles (SiNPs) [2]. SiNPs represent a typical class of engineered nanomaterials and are also one of the major forms of silica. At present, SiNPs have attracted considerable attention due to their large-scale production and extensive application. Owing to their excellent properties, SiNPs are widely used in food processing, synthetic processes, medical diagnostics, and drug delivery, thereby substantially increasing the risk of human exposure [3]. Although the toxicity of crystalline silica has been extensively studied, the toxicity of SiNPs remains largely unknown.

SiNPs can enter the human body through the respiratory tract, digestive tract, and skin, causing damage to the organism, and the lung is the most important and toxicologically affected target organ of SiNPs [3]. Existing studies have demonstrated that SiNPs are closely associated with various pulmonary diseases, including pneumonia, emphysema, pulmonary fibrosis, and even lung cancer, which can be life-threatening in severe cases [4]. However, further studies are still needed on the toxicity of SiNPs both in vitro and in vivo. Alveolar macrophages, as key effector cells in the progression of silicosis, play a central role in initiating and sustaining pulmonary disease-related inflammation [5]. Upon inhalation, silica particles are phagocytosed and cleared by alveolar macrophages that migrate to deposition sites. However, due to the resistance of silica particles to degradation, persistent macrophage activation and continuous release of inflammatory mediators ultimately lead to pulmonary inflammation and fibrosis [6].

STING (also known as TMEM173) is an immune adaptor protein that is involved in regulating signal crosstalk across various physiological and pathological processes [7]. Although it is well-established that STING is transported from the endoplasmic reticulum (ER) to the Golgi apparatus upon recognition of DNA derivatives, emerging evidence indicates that STING can also be transported to other organelles, thereby mediating its immune-dependent (type-I interferon and pro-inflammatory cytokine production) and immune-independent (e.g., autophagy, ER stress, cell death, and lipid metabolism) physiological functions [8]. Upon activation, STING binds and activates tank-binding kinase 1 (TBK1), leading to the dimerization and phosphorylation of interferon regulatory factor 3 (IRF3) [9]. Phosphorylated IRF3 translocates into the nucleus and promotes the transcription and synthesis of type-I interferons [10]. Additionally, STING activates the NF-κB signaling pathway, inducing the synthesis and release of pro-inflammatory cytokines such as IL-6 [11].

Studies have shown that the STING signaling pathway participates in regulating immune responses in various pulmonary inflammatory diseases [10,12]. It has been reported that macrophages produce sphingosine-1-phosphate (S1P) to suppress STING signaling, thereby alleviating inflammation-related vascular injury [13]. Furthermore, pharmacological inhibition of STING effectively reduces IFN-β production and associated tissue damage in mice [14]. Although several studies have explored the relationship between silica exposure and STING signaling, the downstream mechanisms by which STING mediates SiNPs-induced pulmonary inflammation remain unclear.

In this current work, we aim to explore the specific mechanism by which SiNP exposure mediates pulmonary inflammation via the STING signaling pathway, using both in vivo and in vitro experiments, and to further investigate whether NF-κB plays a role in this inflammatory response. Our findings demonstrate that genetic knockout of STING suppresses NF-κB activation, thereby mitigating SiNPs-induced inflammatory responses in macrophages and subsequently ameliorating lung tissue injury and fibrosis in mice. These results suggest that SiNPs may induce pulmonary inflammation via the STING-NF-κB signaling axis.

## 2. Materials and Methods

### 2.1. Particle Preparation and Characterization

Prior to use, amorphous SiNPs (Sigma-Aldrich, St. Louis, MO, USA, #637246) were acid washed in 1 M HCl at 100 °C for 1 h to remove potential endotoxin contamination, washed three times with sterile water, and dried at 200 °C. The removal of endotoxins was confirmed using an Endotoxin Detection Kit (MedChemExpress, Shanghai, China #HY-K1099). The endotoxin level was below the detection limit (<0.005 EU/mL, Figure S1). The actual size, shape, and distribution of SiNPs were determined using transmission electron microscopy (TEM) (Hitachi, Tokyo, Japan, HT7700) and ImageJ 1.52a software. Dynamic light scattering was performed with a Zetasizer (Malvern, Malvern, UK, Zetasizer Nano ZS90) to determine the hydrodynamic size and zeta potential of SiNPs in various dispersion media. Before being applied to cells or animals, the SiNP suspension was sonicated for 15 min using a bath sonicator (Skymen, Shenzhen, China, JP-080S) to minimize aggregation.

### 2.2. Animal Maintenance and Exposure

Wild-type (WT) C57BL/6 mice and STING-deficient (STING^−/−^) mice were obtained from GemPharmatech (Nanjing, China) and bred in the Medical Laboratory Animal Center of Shandong Second Medical University. The mice were randomly allocated to groups using a random number table. All animal experiments were approved by the Ethics Committee of the Laboratory Animal Administration of Shandong Second Medical University in Shandong Province under number 2023SDL231. For experiments, adult (male, 8-week-old, 20–22 g, *n* = 6 per group) animals were freely provided with food and water under standard breeding conditions, with a 12 h reversed light/dark cycle and controlled temperature (22 ± 2 °C).

Mice received a single intranasal instillation of 40 µL of either SiNPs saline suspension (1 mg/mouse) or saline vehicle. After 7 days, the mice were euthanized by intraperitoneal injection of 150 mg/kg sodium pentobarbital. The dosage and exposure duration were adapted from the previously published protocol that has been proven to cause acute lung injury [12]. The left lungs were fixed in 4% paraformaldehyde for at least 72 h, followed by paraffin embedding and subsequent histological analysis. The other parts of the lung tissue were stored at −80 °C for Western blot and real-time quantitative PCR (RT-qPCR) experiments.

### 2.3. BMDM Extraction and Culture

BMDMs were prepared as described previously with some modifications [12]. Briefly, C57BL/6 mice were sacrificed, and the femur and tibia bones were collected. Then bone marrow cells were obtained by flushing the bone marrow cavity with DMEM medium (Keygen Biotech, Nanjing, China), followed by filtration through a 70 μm cell filter. The cells were resuspended and cultured in complete DMEM (Keygen Biotech, China) supplemented with 10% FBS (Excell, Suzhou, China), 100 U/mL penicillin and 100 µg/mL streptomycin (Beyotime Biotechnology, Shanghai, China), and 30% (*v*/*v*) L929 conditioned medium as a source of M-CSF for a 7-day differentiation period at 37 °C in a humidified incubator containing 5% CO_2_. Following differentiation, cells (3 × 10^6^/mL) were washed and maintained in fresh medium for an additional 3 days prior to subsequent experiments. Mature BMDMs exhibited typical polygonal or spindle-shaped morphology.

### 2.4. Cell Viability and Lactate Dehydrogenase (LDH) Release Assay

Cell injury was evaluated by measuring cell viability and LDH release. Approximately 8000 BMDMs were seeded in 96-well plates. After SiNP exposure (25 μg/mL, 12 h), the supernatant was collected and retained for subsequent LDH detection. The cells were then incubated for approximately 2 h with the detection reagent prepared from fresh medium containing 10% CCK-8 reagent (Selleck, Houston, TX, USA, #B34302). The absorbance was measured at 450 nm using a microplate reader (Agilent BioTek, Santa Clara, CA, USA). Intracellular LDH release into the culture medium serves as an indicator of irreversible cell death resulting from damaged cell membranes. The release of LDH was measured using a commercial assay kit (Sangon, Shanghai, China, #D799208) to evaluate the effect of SiNPs on cell membrane integrity. At the end of SiNP exposure (25 μg/mL, 12 h), the culture medium was centrifuged to collect the supernatant. The LDH activity in the supernatant was then analyzed according to the manufacturer’s instructions by measuring the absorbance at a wavelength of 450 nm. The amount of LDH released is expressed as the LDH activity (U/mL).

### 2.5. Western Blot Analysis

Protein extraction was performed as previously described [15]. The lung tissues and BMDMs were lysed with 1× RIPA lysis buffer [150 mM sodium chloride, 50 mM Tris-HCl (pH 7.4), 1% NP-40, 0.1% SDS, 0.5% sodium deoxycholate, 1× EDTA-free protease inhibitor cocktail (Selleck, USA), and 1× phosphatase inhibitor cocktail (Selleck, USA)]. After centrifugation at 4 °C, 12,000 *g* for 10 min, the total protein concentration in the supernatant was quantified by a BCA assay kit (Dingguo Biotech, Beijing, China). Proteins (30–50 µg per sample) were separated by SDS-polyacrylamide gel electrophoresis (SDS-PAGE) and then transferred onto nitrocellulose membranes. Membranes were blocked with 5% non-fat milk at room temperature for 2 h. Then the membranes were incubated with the appropriate primary antibody overnight at 4 °C on a shaker. The following primary antibodies were used: STING (#50494, Cell Signaling Technology, Danvers, MA, USA), cGAS (#31659, Cell Signaling), TBK1 (#3504, Cell Signaling), p-TBK1 (#5483, Cell Signaling), p-p65 (#bs-0982R, Bioss, Beijing, China), p65 (#F0006, Selleck), Lamin B (#12987-1-AP, Proteintech, Wuhan, China), and β-actin (#D110001, Sangon). After washing in 1× TBST three times, the membranes were further incubated with HRP-conjugated secondary antibodies (Sangon, China) for an additional 1 h at room temperature. Protein bands were visualized using Super ECL Detection Reagent (Yeasen, Shanghai, China). Representative blots were chosen from at least three independent experiments, and the protein expression levels were calculated by ImageJ software. β-actin was used as a housekeeping protein.

### 2.6. RNA Extraction and RT-qPCR

Gene expression levels were measured by RT-qPCR. Total RNA was extracted from BMDMs and lung tissues using TRNzol Universal reagent (TIANGEN, Beijing, China). For cell samples, 500 μL of TRNzol was added. For tissue samples, 500 μL of TRNzol was added, and samples were homogenized using a cryogenic grinder. Chloroform (0.1 mL) was then added to each sample, vortexed for 15 s, left at room temperature for 3 min, and centrifuged at 12,000 *g* for 15 min at 4 °C. The upper aqueous phase was transferred to a new tube, mixed with an equal volume of isopropanol, and incubated for 10 min. After centrifugation at 12,000 *g* for 10 min at 4 °C, the supernatant was removed, and the RNA pellet was retained. The pellet was then washed once with 75% ethanol. After air-drying, the RNA was dissolved in DEPC water. RNA concentration and purity were measured using a NanoDrop spectrophotometer (Thermo Fisher Scientific, Waltham, MA, USA). The A260/A280 ratio should fall within the range of 1.8 to 2.1. The purified RNA was reverse transcribed into cDNA using Hifair^®^ III 1st Strand cDNA Synthesis SuperMix (Yeasen, China). RT-qPCR was performed on diluted cDNA with 2× SYBR Green qPCR Master Mix (Selleck, USA) to quantify the mRNA expression levels of different targeted proteins. Primers used for RT-qPCR are listed in Table S2. Relative gene expression levels from three independent experiments were calculated using the 2^−△△Ct^ method, and GAPDH was used as the internal control.

### 2.7. Nuclear and Cytoplasmic Protein Extraction

The BMDMs were washed with PBS three times, then corresponding proteins were prepared using a Nuclear Protein Extraction Kit (Solarbio, Beijing, China) according to the manufacturer’s protocol and our previous publication [16]. Briefly, 5 × 10^6^ cells were trypsinized, collected, centrifuged, and washed with PBS. Then, cells were resuspended in the extraction solution for 20 min. After centrifugation at 2000 *g* for 5 min, the supernatant (cytoplasmic fraction) was transferred to another pre-cooled centrifuge tube. The pellet was washed and then lysed to obtain nuclear protein. The protein concentrations were measured using the BCA method.

### 2.8. Immunofluorescence Assay of NF-κB p65 Nuclear Translocation

After exposure to SiNPs for 6 h, BMDMs were washed with PBS three times and fixed with freshly prepared 4% paraformaldehyde for 20 min, permeabilized in 0.1% Triton X-100 for 10 min, followed by blocking incubation in 3% BSA for 1 h. Then, BMDMs were incubated with p65 antibody (#F0006, Selleck) overnight at 4 °C, and then incubated with a secondary Cy3-conjugated antibody (#SA00009-2, Proteintech) for an additional 1 h at room temperature. DAPI (MedChemExpress, USA) was incubated in a dark environment for 15 min. TNFα (Yeasen, China) was used as the positive control (10 ng/mL, 1 h). Finally, the images were obtained under a fluorescence microscope (OLYMPUS, Tokyo, Japan, BX53F2C).

### 2.9. Histological Analysis

Histopathological analysis was performed following the standard laboratory procedures. For each group, three mice were used to prepare paraffin-embedded sections, and 3 to 5 microscopic fields were captured per section. Briefly, the embedded paraffin lung tissue blocks were sectioned into 4 μm-thick slices, which were then mounted on glass slides. Hematoxylin and eosin (H&E) staining and Masson staining (Servicebio, Wuhan, China) were carried out according to the manufacturer’s instructions to evaluate pathological changes in lung tissue (inflammation and fibrosis). The images were captured using a microscope (OLYMPUS, BX53F2C).

### 2.10. Immunohistochemistry Assay

Immunohistochemistry was used to detect pulmonary macrophage infiltration. For each group, three mice were used to prepare paraffin-embedded sections, and 3 to 5 microscopic fields were captured per section. Briefly, after dewaxing and antigen retrieval of paraffin sections, endogenous peroxidase was blocked with 3% hydrogen peroxide solution. Sections were blocked with 3% bovine serum albumin (BSA) (Sangon, China) for 1 h and then incubated with F4/80 primary antibody (GB113373, Servicebio) at 4 °C overnight. Then, the sections were incubated with secondary antibody at 37 °C for 30 min. Diaminobenzidine (DAB) (Servicebio, China) was used as the chromogen, followed by hematoxylin counterstaining to visualize cell nuclei. The sections were imaged by a microscope (OLYMPUS, BX53F2C). ImageJ was employed to analyze the area ratio occupied by positive cells in the images.

### 2.11. Statistical Analysis

The results are represented as the mean ± standard deviation (SD). Statistical analysis was performed using GraphPad Prism 8.4.0. Shapiro–Wilk test was applied for the normality test. One-way ANOVA followed by Tukey’s multiple comparison test was utilized in intergroup comparisons of more than two groups. For experiments involving two independent variables, two-way ANOVA with Tukey’s multiple comparison test was applied. *p* < 0.05 was considered statistically significant.

## 3. Results

### 3.1. Characterization of SiNPs

**Figure 1:**
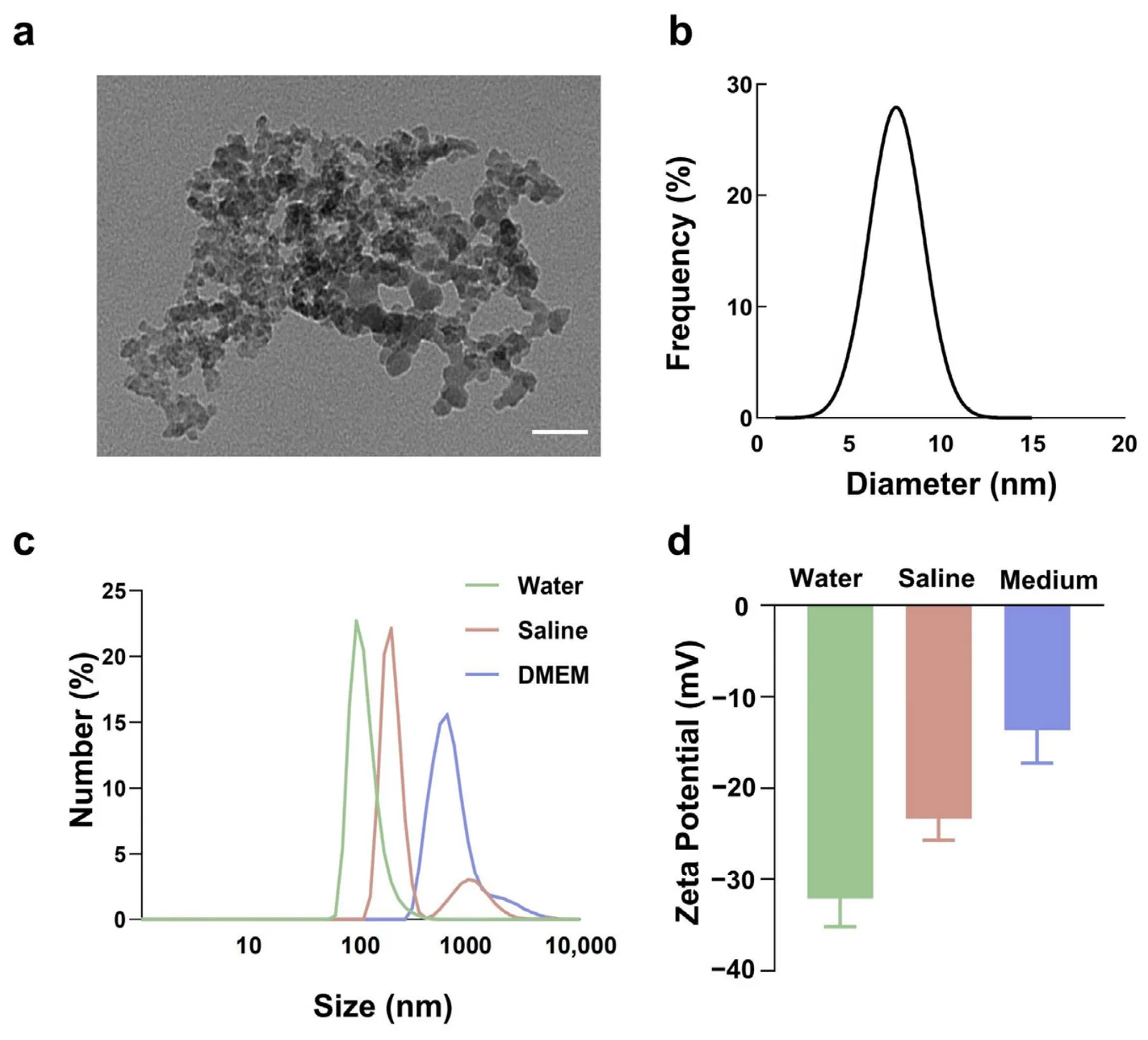
Characterization of SiNPs. (**a**) TEM image of SiNPs. Scale bar: 50 nm. (**b**) Diameter distribution of SiNPs taken by TEM. SiNPs hydrated particle size (**c**) and Zeta potential (**d**) of SiNPs in water, saline, or medium containing 10% FBS.

Prior to the toxicity investigation, the SiNPs were characterized. The morphology (Figure 1a) and actual size (Figure 1b) of SiNPs were measured by TEM. The chosen SiNPs were non-aggregated, near-spherical particles with sizes ranging predominantly from 5 to 15 nm. Additionally, to assess the potential influence of the dispersion media on SiNPs, we subsequently measured the hydrodynamic sizes (Figure 1c) and zeta potentials (Figure 1d) of SiNPs in water, physiological saline, and DMEM complete culture medium (Table S1). The results revealed that the hydrodynamic size of SiNPs in the dispersion media was larger than their original size. Zeta potential serves as a marker of colloidal stability, where a higher absolute value generally indicates greater resistance to aggregation. As shown in Figure 1d, SiNPs exhibited relatively favorable stability in water. However, their stability was compromised in saline solution and culture medium, indicating SiNPs were prone to aggregation.

### 3.2. SiNPs Activate STING Signaling Pathway

**Figure 2:**
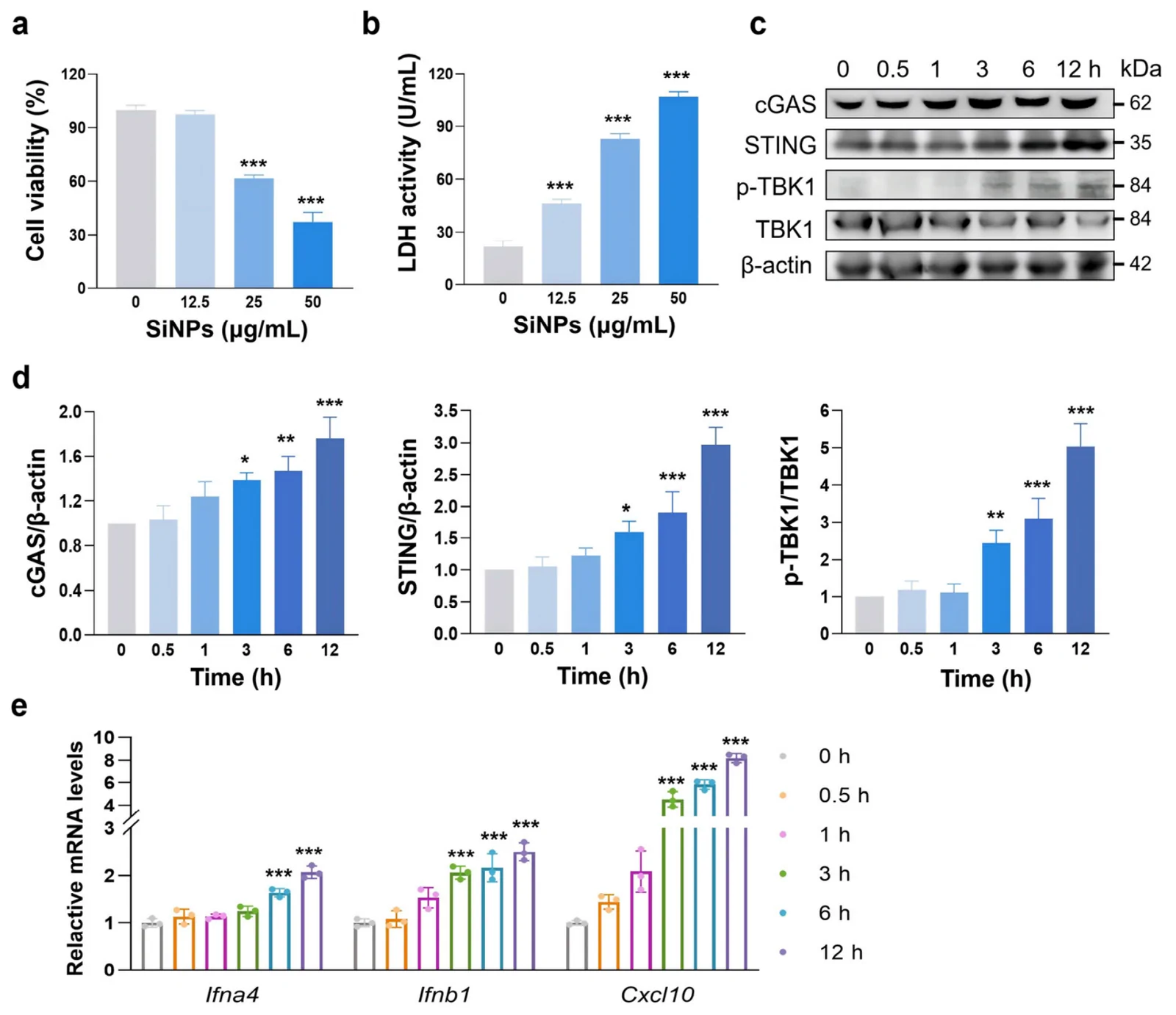
Activation of the STING signaling pathway by SiNPs in BMDMs. Cell viability (**a**) and LDH release (**b**) of BMDMs were measured after treatment with different concentrations of SiNPs for 12 h. LDH release in the supernatant indicated BMDM injury. (**c**) Immunoblots of cGAS, STING, p-TBK1, and TBK1 in BMDMs with β-actin as a reference. BMDMs were exposed to 25 μg/mL SiNPs for the indicated durations (0–12 h). (**d**) Quantitative analysis of STING, cGAS, and p-TBK1/TBK1 by Immunoblots. (**e**) Time-dependent transcriptional induction of *Ifna4*, *Ifnb1*, and *Cxcl10* measured by RT-qPCR after SiNP exposure for the indicated durations (0–12 h). Data represent fold-changes relative to the untreated control, normalized to GAPDH (mean ± SD). Data from three independent experiments were expressed as the means ± SD, and statistical analysis was performed via one-way ANOVA. * *p* < 0.05, ** *p* < 0.01, *** *p* < 0.001 compared with the control group. (Original Western Blot images see Supplementary Figure S3).

During the process of lung injury induced by silica particles, macrophages have the ability to engulf silica particles and secrete different cytokines to trigger multiple pulmonary stress responses [17]. Meanwhile, macrophages serve as typical host cells expressing STING [12]. Therefore, we selected BMDMs as the in vitro model. Initially, we evaluated the cytotoxic effect of SiNPs on BMDMs. As shown in Figure 2a, SiNP exposure resulted in a concentration-dependent decrease in the cell viability of BMDMs, while concurrently promoting the release of intracellular LDH (Figure 2b). Western blot analysis demonstrated that SiNPs significantly upregulated the expression of cGAS and STING, as well as the phosphorylation level of TBK1 in BMDMs (Figure 2c,d). The mRNA expression levels of downstream target molecules in the STING signaling pathway, including interferon α4 (Ifna4), interferon β1 (Ifnb1), and C-X-C motif chemokine ligand 10 (Cxcl10), were also significantly increased (Figure 2e and Figure S2). These results indicate that the STING signaling pathway in BMDMs is activated after exposure to SiNPs.

### 3.3. SiNP Exposure Causes an Inflammatory Response in BMDMs via STING

**Figure 3:**
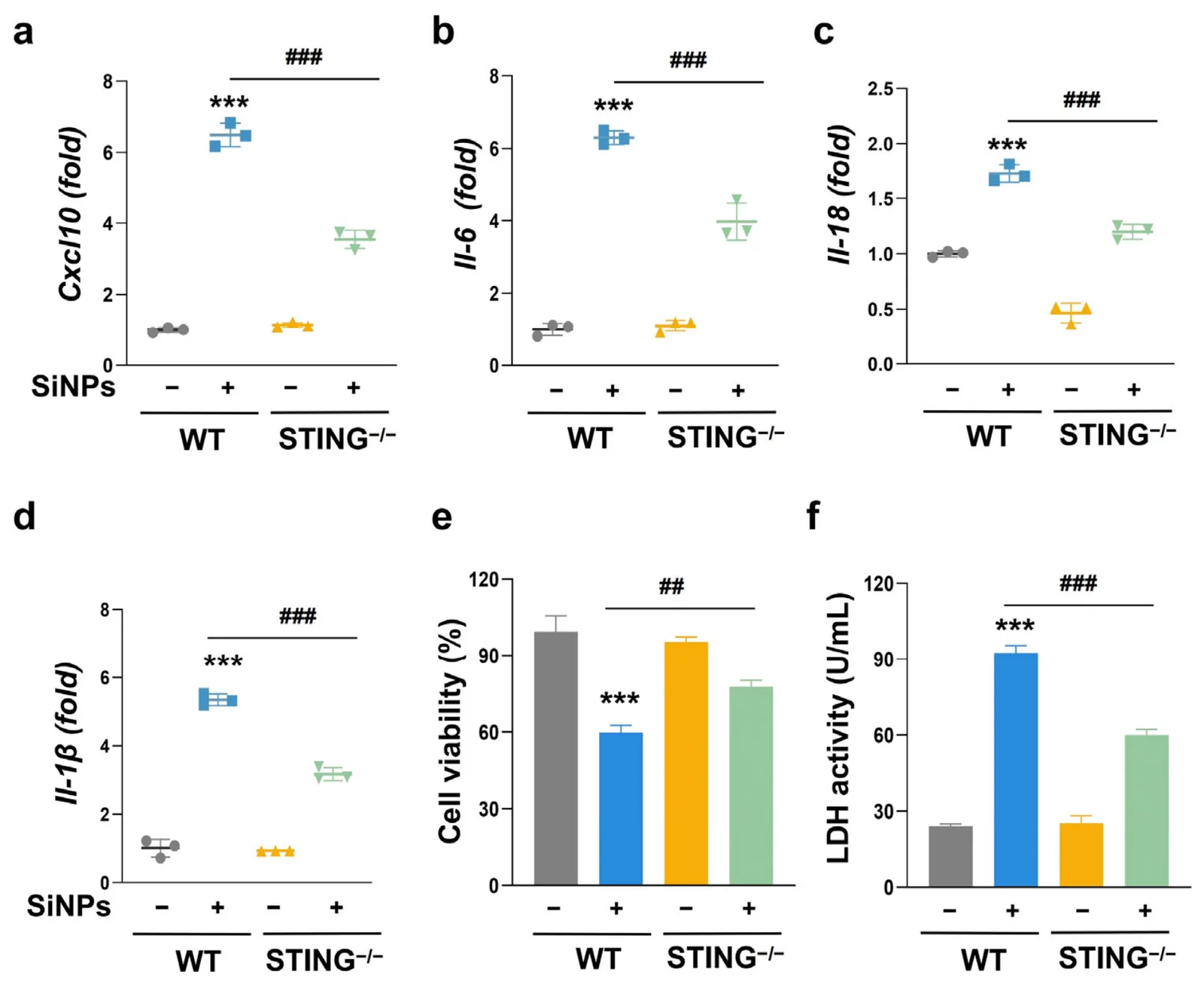
STING-dependent inflammatory activation in BMDMs following SiNP exposure. (**a**–**d**) BMDMs from wild-type (WT) or STING-deficient (STING^−/−^) mice were unstimulated or stimulated with SiNPs (25 µg/mL) for 6 h. *Cxcl10*, *Il-6*, *Il-18*, and *Il-1β* transcripts were measured by RT-qPCR. Data represent fold changes relative to the untreated control, normalized to GAPDH; cell viability (**e**) and LDH release (**f**) of WT and STING^−/−^ BMDMs were measured after 25 μg/mL SiNP exposure for 12 h. Data from three independent experiments were expressed as the means ± SD, and statistical analysis was performed via two-way ANOVA *** *p* < 0.001 compared with the WT control group (SiNPs vs. untreated control in WT BMDMs); ## *p* < 0.01, ### *p* < 0.001 compared with the WT SiNPs-exposed group (STING^−/−^ + SiNPs vs. WT + SiNPs).

Previous studies have demonstrated that SiNPs can trigger pulmonary inflammation [18], and the STING pathway has been shown to play an essential role as a mediator of inflammation in the settings of cellular stress and tissue injury [10]. Therefore, to investigate the inflammatory effects of SiNPs on BMDMs and the potential involvement of the STING pathway, we isolated BMDMs from STING-knockout (STING^−/−^) mice. We first examined the expression of *Cxcl10*, a downstream gene of STING. As shown in Figure 3a, SiNPs-induced *Cxcl10* expression was significantly suppressed following STING knockout, confirming the effective blockade of the STING signaling pathway. IL-6, IL-1β, IL-8, etc., are known as the key inflammatory mediators [19]. Exposure to SiNPs caused the overexpression of the *Il-6*, *Il-18*, and *Il-1β* genes in BMDMs. Notably, genetic ablation of STING attenuated SiNPs-induced elevation in the expression levels of these inflammatory cytokines (Figure 3b–d). Subsequent experiments further demonstrated that STING deficiency effectively alleviated the cell death and LDH release caused by SiNPs (Figure 3e,f). Collectively, these results indicate that SiNP exposure triggers an inflammatory response in BMDMs through the STING pathway.

### 3.4. SiNPs Activate NF-κB via STING Signaling Pathway

**Figure 4:**
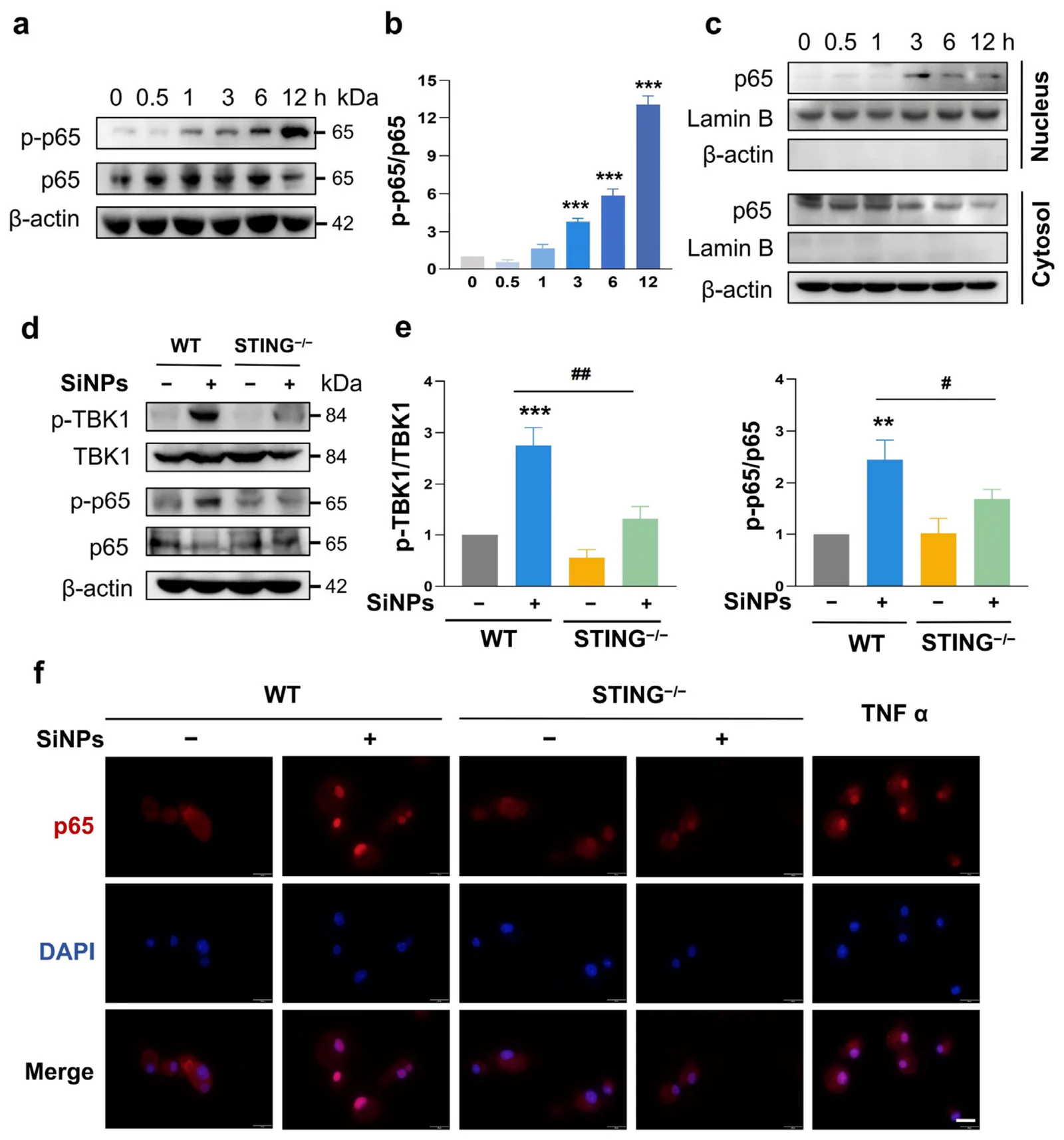
SiNPs activate NF-κB p65 via the STING signaling pathway. (**a**) Immunoblots of p-p65 and p65 in BMDMs with β-actin as a reference. BMDMs were exposed to SiNPs (25 μg/mL) for the indicated times. (**b**) Quantitative analysis of p-p65/p65 by Immunoblots. Data from three independent experiments were expressed as the means ± SD, and statistical analysis was performed via one-way ANOVA. *** *p* < 0.001 compared with the control group. (**c**) Western blot for NF-κB p65 expression in cytoplasmic and nuclear fractions of BMDMs exposed to SiNPs. (**d**) Immunoblots of p-TBK1, TBK1, p65, and p-p65 in wild-type (WT) and STING^−/−^ BMDMs with β-actin as a reference. BMDMs were exposed to 25 μg/mL SiNPs for 12 h. (**e**) Quantitative analysis of p-p65/p65 and p-TBK1/TBK1 by Immunoblots. (**f**) Immunofluorescence staining of p65 (red) and nuclei (blue) was detected, and merged images indicated the distribution of p65 after SiNP exposure for 6 h. TNFα was used as the positive control. Scale bar: 20 μm. Data from three independent experiments were expressed as the means ± SD, and statistical analysis was performed via one-way ANOVA (**b**) and two-way ANOVA (**e**). ** *p* < 0.01, *** *p* < 0.001 compared with the WT control group (SiNPs vs. untreated control in WT BMDMs); # *p* < 0.05, ## *p* < 0.01 compared with the WT SiNPs-exposed group (STING^−/−^ + SiNPs vs. WT + SiNPs). (Original Western Blot images see Supplementary Figure S3).

We have confirmed that SiNPs induce BMDM death and activate the STING pathway, leading to increased secretion of inflammatory cytokines. Previous studies have reported that STING activation can subsequently trigger the NF-κB pathway through TBK1 recruitment [20,21,22], and NF-κB serves as a key regulator of inflammatory cytokine expression [23]. Therefore, we next investigated whether SiNPs activate the NF-κB pathway and its potential relationship with STING signaling. To clarify the effect of SiNPs on the NF-κB pathway, BMDMs were exposed to SiNPs in a time-dependent manner. Western blot analysis showed that phosphorylation of the NF-κB subunit p65 (p-p65) increased progressively with prolonged SiNP exposure (Figure 4a), and quantitative analysis verified this time-dependent trend (Figure 4b). As nuclear translocation of p65 represents a hallmark of NF-κB activation, we next extracted nuclear and cytoplasmic proteins to analyze p65 distribution. With increasing SiNP treatment time, cytoplasmic p65 decreased while nuclear p65 increased (Figure 4c), indicating SiNPs effectively activated the NF-κB pathway. Furthermore, STING deficiency significantly attenuated SiNPs-induced p65 phosphorylation (Figure 4d,e) and markedly suppressed p65 nuclear translocation, as evidenced by immunofluorescence analysis (Figure 4f). In conclusion, our findings demonstrate that SiNPs activate the NF-κB pathway through STING signaling.

### 3.5. SiNPs Induce Inflammation Through the STING-NF-κB Signaling Pathway

**Figure 5:**
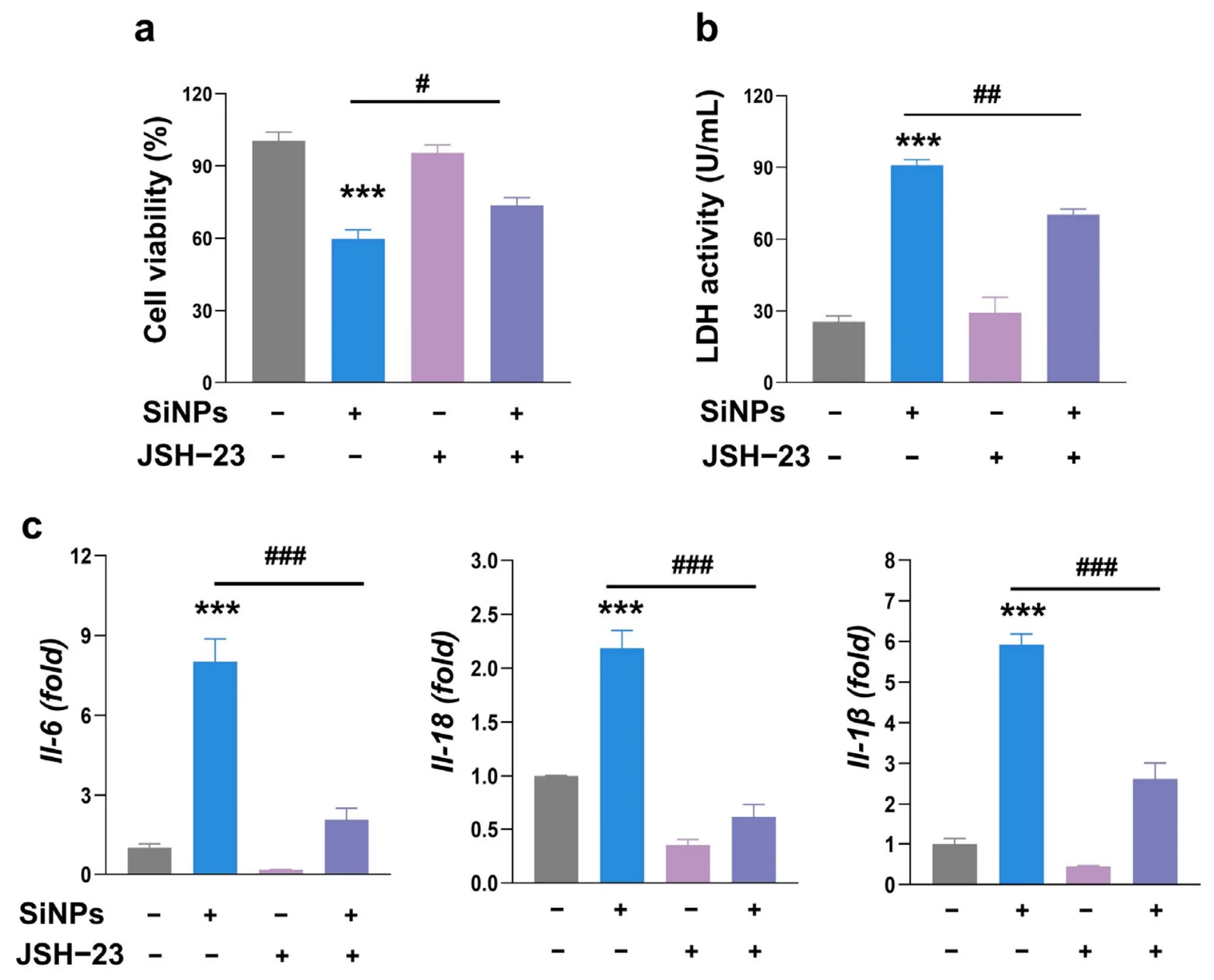
SiNPs trigger inflammatory responses via the STING-NF-κB p65 signaling axis. Cell viability (**a**) and LDH release (**b**) of BMDMs were determined following exposure to 25 μg/mL SiNPs for 12 h with or without JSH-23 (10 μM, NF-κB transcriptional activity inhibitor). (**c**) Effect of the NF-κB inhibitor on SiNPs-induced *Il-6*, *Il-18* and *Il-1β* mRNA expression. BMDMs were pre-treated with or without JSH-23 for 1 h, followed by SiNP exposure for 6 h. Data from three independent experiments were expressed as the means ± SD, and statistical analysis was performed via two-way ANOVA. *** *p* < 0.001 compared with the control group. # *p* < 0.05, ## *p* < 0.01, ### *p* < 0.001 compared with the SiNPs alone group (JSH-23 + SiNPs vs. SiNPs alone).

We next investigated whether the induction of inflammatory cytokines by SiNPs via the STING pathway is dependent on the NF-κB pathway. BMDMs were treated with the NF-κB inhibitor JSH-23 prior to SiNP exposure. Our results demonstrated that NF-κB pathway inhibition attenuated the SiNPs-induced decrease in cell viability (Figure 5a) and reduced LDH release (Figure 5b). Additionally, the gene expression levels of *Il-6*, *Il-18*, and *Il-1β* induced by SiNPs were significantly downregulated following JSH-23 treatment (Figure 5c). These findings collectively suggest that SiNPs induce inflammatory responses through the STING-NF-κB signaling pathway, providing crucial mechanistic insights into SiNPs-induced inflammatory responses.

### 3.6. SiNP Exposure Causes Inflammatory Infiltration and Pulmonary Fibrosis in Mice

**Figure 6:**
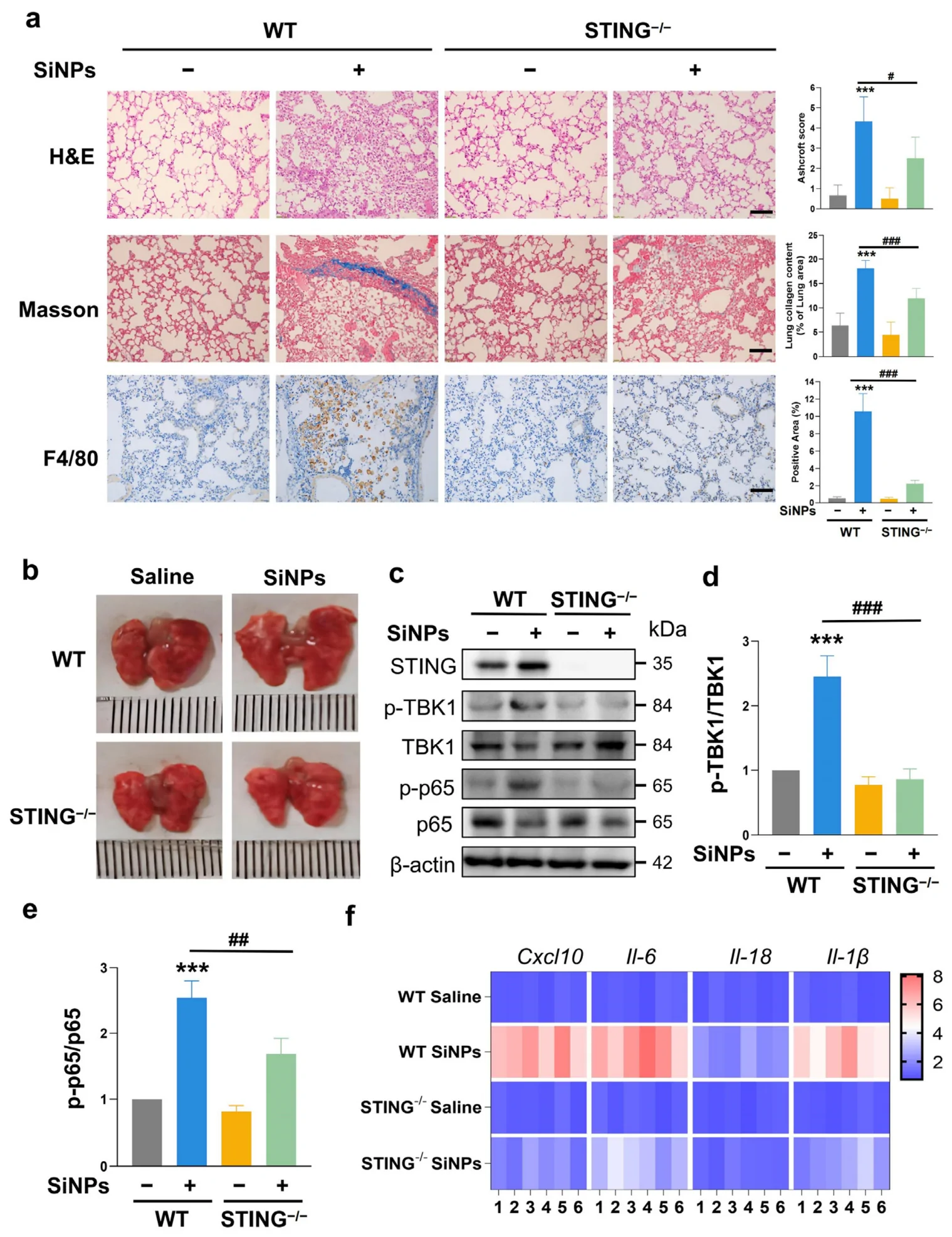
STING-dependent pulmonary inflammatory infiltration triggered by SiNPs in mice. (**a**) H&E, Masson, and F4/80 representative images of lungs following SiNP exposure in WT or STING^−/−^ mice. Scale bar: 100 µm. Quantitative analyses of Ashcroft scores, collagen-positive area percentage, and F4/80-positive area percentage are shown in the bar graphs (right panels). (**b**) Morphological alterations in pulmonary tissues across experimental groups. (**c**) Western blot analysis of p-TBK1, TBK1, p-p65, and p65 expression in lungs of WT and STING^−/−^ mice with β-actin as a reference. (**d**,**e**) Quantitative analysis of p-TBK1/TBK1 and p-p65/p65 by Western blot. Data from three independent experiments were expressed as the means ± SD, and statistical analysis was performed via two-way ANOVA. (**f**) *Cxcl10*, *Il-6*, *Il-18* and *Il-1β* mRNA expression in lungs of WT and STING^−/−^ mice. Data represent fold-changes relative to the untreated control, normalized to GAPDH. *** *p* < 0.001 compared to the WT control group. # *p* < 0.05, ## *p* < 0.01, ### *p* < 0.001 compared with the WT SiNPs-exposed group (STING^−/−^ + SiNPs vs. WT + SiNPs). (Original Western Blot images see Supplementary Figure S3).

After confirming the cell damage caused by SiNPs in vitro, the lung injury was further evaluated in a murine model following exposure to SiNPs via intranasal instillation. The pulmonary pathological results of H&E staining (Figure 6a) revealed that WT mice in the SiNP exposure group exhibited marked alveolar septa thickening, inflammatory cell infiltration into the alveoli, and disrupted alveolar architecture. Conversely, lung tissues from STING^−/−^ mice treated with SiNPs displayed significantly reduced inflammatory infiltration and relatively orderly alveolar structures. Masson staining results demonstrated extensive deposition of blue-stained collagen fibers in the lung tissues of WT mice in the SiNP exposure group. Ashcroft scoring revealed that SiNP exposure significantly increased the pulmonary fibrosis score in WT mice compared with the saline control group (from 0.67 ± 0.52 to 4.33 ± 1.21), whereas STING knockout significantly reduced it to 2.50 ± 1.05. Collectively, the Ashcroft score and the quantitative analysis of collagen area further demonstrated that STING knockout effectively alleviated SiNP exposure-induced pulmonary fibrosis. Immunohistochemical staining for F4/80 (a macrophage marker) showed a large accumulation of macrophages in the lungs of WT mice exposed to SiNPs, whereas the F4/80-positive area was significantly decreased in the STING-deficient SiNPs-exposed group, suggesting that STING knockout inhibited SiNPs-induced macrophage infiltration. Moreover, the captured images visually demonstrated that STING knockout alleviated the swelling of lung tissues induced by SiNPs (Figure 6b).

**Figure 7:**
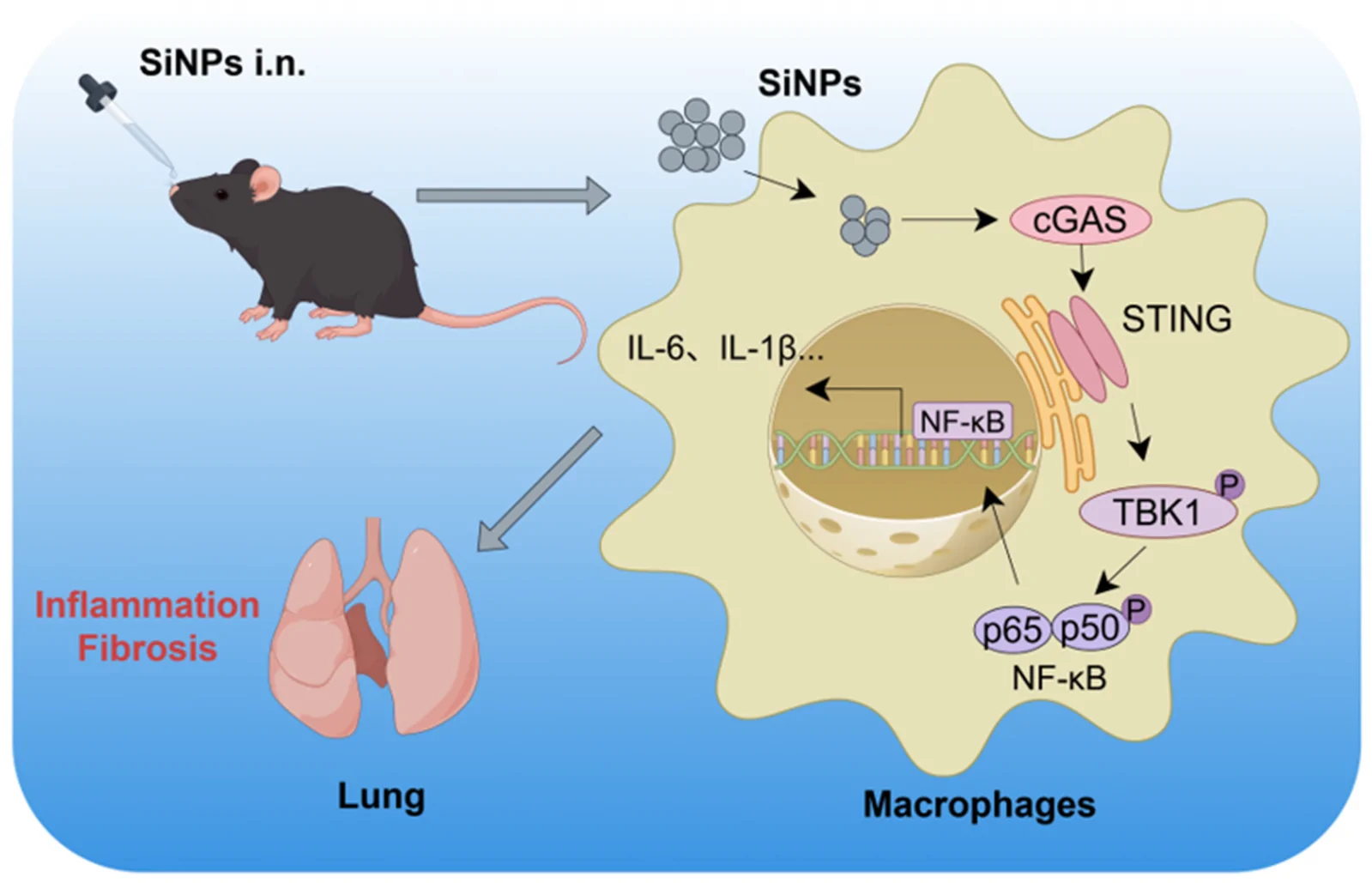
Schematic illustration of how SiNPs induce pulmonary inflammation and fibrosis by activating the STING-NF-κB signaling axis. SiNPs are inhaled and taken up by pulmonary macrophages. This triggers the activation of the STING pathway, which subsequently promotes the phosphorylation of TBK1 and NF-κB p65. The activated NF-κB then translocates into the nucleus to drive the expression of pro-inflammatory cytokines, ultimately leading to lung inflammation and fibrosis. The figure was drawn by Figdraw (www.figdraw.com).

In further in vivo mechanistic investigations, consistent with in vitro experiments, SiNP exposure activated the STING-NF-κB signaling pathway in the lungs, characterized by a significant increase in the phosphorylation levels of TBK1 and p65. The levels of p-TBK1 and p-p65 in STING^−/−^ mice after SiNP exposure were significantly reduced, indicating that STING deficiency blocked the activation of the TBK1-NF-κB signaling pathway (Figure 6c–e). We further detected the mRNA levels of inflammatory cytokines in lung tissues (Figure 6f), and the results showed that STING deficiency alleviated the significant upregulation of mRNA expression of *Cxcl10*, *Il-6*, *Il-18*, and *Il-1β* induced by SiNPs. In summary, SiNP exposure induced inflammatory infiltration, fibrosis, and abnormal expression of multiple inflammatory cytokines in lung tissues by activating the STING-NF-κB signaling pathway (Figure 7).

## 4. Discussion

SiNPs are widely used in biomedical applications, cosmetics production, and general industrial processes, raising significant concerns regarding their biosafety [3,24,25]. The selected SiNPs primarily exhibit a particle size range of 5–15 nm. Particles smaller than 100 nm can be internalized by all cell types via endocytosis, having a greater chance of transporting across biological barriers and interacting with immune cells in the blood, particularly macrophages, resulting in an immune response [26,27]. After entering the blood, nanoparticles interact with biomolecules to form a protein corona, which can mask or alter the surface properties of the nanoparticles and influence subsequent biological responses [28]. In this study, the measured hydrodynamic diameter of SiNPs in complete medium was larger than that in aqueous solution, suggesting the formation of a protein corona.

Although several studies have demonstrated that SiNPs elicit pulmonary inflammation and involve the STING signaling pathway, the underlying molecular mechanisms are not completely understood [12,29]. Our study demonstrates that NF-κB is a key mediator in SiNPs-induced pulmonary inflammation and injury mediated by the activation of the STING signaling pathway. It is generally accepted that SiNP exposure leads to pathological features including pulmonary fibrosis, mucus accumulation, and inflammatory cell infiltration [30,31,32]. Our experimental model exhibits characteristics consistent with those reported in prior investigations, confirming its suitability as a reliable tool for investigating SiNPs-induced pulmonary inflammation.

Recently, several studies have shown that silica exposure activates the STING pathway [12,17,33]. As an essential component of the innate immune system, the STING pathway plays a key role in triggering inflammation and promoting the expression of inflammatory cytokines [34]. Upon activation, STING recruits and phosphorylates TBK1 molecules to form a STING-TBK1 signalosome, resulting in initiation of IRF3 phosphorylation and finally causing type-I interferon expression [35]. In addition, STING promotes the phosphorylation of IκB, an inhibitor of NF-κB, leading to IκB degradation by the ubiquitin-proteasome pathway and promoting NF-κB nuclear translocation [11]. NF-κB regulates the transcription of inflammatory cytokines, and its activation induces a prominent pro-inflammatory cytokine response [36]. In our study, SiNP exposure activates the STING pathway in BMDMs, promoting the nuclear translocation of NF-κB and upregulating the gene expression of inflammatory cytokines. However, in STING^−/−^ mice, SiNPs-induced inflammation was alleviated, indicating that the STING signaling pathway regulates SiNPs-induced inflammatory responses.

Previous studies have shown that TBK1 acts as a common upstream kinase mediating both IRF3 and NF-κB signaling [21]. However, emerging evidence indicates that IRF3 also plays a regulatory role in the NF-κB signaling pathway. There is evidence suggesting that IRF3, as an adaptor of immune signaling, engages with STING at Ser358 and recruits TRAF6, resulting in the activation of NF-κB [37]. While these studies suggest potential crosstalk between IRF3 and NF-κB, the present study focuses on the STING-TBK1-NF-κB axis, and the possible involvement of IRF3 in SiNPs-induced NF-κB activation remains to be further investigated. Fischer et al. reported that the activation of STING promotes the localization of the linear ubiquitin chain assembly complex E3 ligase (HOIP) to the LC3B-associated Golgi membrane, synthesizing M1-Ub chains to stimulate the NF-κB pathway [38]. Zhang et al. found that the NF-κB pathway also enhances STING signaling by inhibiting STING trafficking to lysosomes for degradation and causing ligand-independent STING autoactivation [23]. These findings highlight the extensive crosstalk between the STING and NF-κB signaling networks. Therefore, further studies are needed to elucidate the interaction and biological significance of the STING-NF-κB axis.

The limitations of our study should be acknowledged. All animal experiments were carried out exclusively in male mice, and whether our findings can be extended to female subjects remains to be determined. Further validation studies incorporating female mice would help address this question. We employed a single 7-day endpoint following SiNP instillation, which may not fully capture the dynamic progression of fibrotic changes. In addition, although we observed upregulation of cGAS protein expression following SiNP exposure, we did not determine whether SiNP-induced STING activation is mechanistically dependent on cGAS, and the upstream signals responsible for this process remain to be elucidated. As such, our study focuses on the downstream STING–NF-κB axis rather than the complete cGAS-STING cascade. It is also worth noting that the dose used in this study (1 mg/mouse) was adopted based on a previously established acute exposure protocol [12], which has been widely used for mechanistic investigation of silica-induced pulmonary injury. In animal studies, high-dose exposure is often employed to model the pathological processes resulting from long-term low-dose exposure in humans, rather than to directly simulate actual occupational exposure doses. While this acute exposure model permits the observation of early pathogenic events under controlled conditions, it does not perfectly mirror the repeated, long-term exposures typical of occupational environments like mines. Thus, applying our results directly to chronic exposure situations calls for some caution, and more work is needed to explore the effects of sustained silica exposure.

## 5. Conclusions

Our study elucidates the mechanisms of lung inflammation induced by small-sized silica nanoparticles. SiNP exposure activates the STING-TBK1-NF-κB axis, triggering the release of inflammatory cytokines. Both knockout of STING and pharmacological inhibition of NF-κB alleviated the SiNPs-induced inflammatory response in BMDMs. We also established a lung injury model induced by SiNPs in mice, in which STING knockout suppressed pulmonary fibrosis and inflammatory infiltration, as shown by H&E staining, Masson staining, and F4/80 immunohistochemistry. Further in vivo experiments confirmed that SiNPs induce lung inflammation via the STING-NF-κB signaling pathway. Our findings provide new insights and potential therapeutic targets for the treatment of silica particles-induced silicosis.

## Data Availability

The original contributions presented in this study are included in the article/Supplementary Materials. Further inquiries can be directed to the corresponding author.

## Supplementary Materials

The following supporting information can be downloaded at: https://www.mdpi.com/article/10.3390/biom16091273/s1, Figure S1: Endotoxin level of SiNPs; Figure S2: Ifna4, Ifnb1 and Cxcl10 transcripts measured by real-time PCR; Figure S3: Original Western Blot Images. Table S1: Summary of the physical properties of SiNPs; Table S2: Primer Sequences.
