# Supplementary material for: Silica Nanoparticles Elicit Pulmonary Inflammation via STING-Dependent Activation of NF-κB p65 Signaling Pathway
Source: Biomolecules. 2026 Sep 3;16(9):1273. doi: 10.3390/biom16091273 (PMC13604276; doi:10.3390/biom16091273)
Supplement: Supplementary file 1 [file biomolecules-16-01273-s001.zip › biomolecules-4478988-supplementary.pdf]

Silica Nanoparticles Elicit Pulmonary Inflammation via STING-Dependent Activation of NF-κB p65 Signaling Pathway

Junyi Gao [1,†], Fei Wu [1,†], Bing Li [1], Yuhang Ji [1], Hongxu Dong [1], Dongfeng Wang [2] and Zixuan Liu [1,*]

[1] School of Life Science and Technology, Shandong Second Medical University, Weifang 261053, China; gaojunyi@stu.sdsmu.edu.cn (J.G.); wufei@sdsmu.edu.cn (F.W.); libing20230052@stu.sdsmu.edu.cn (B.L.); 20247440007ji@stu.sdsmu.edu.cn (Y.J.); dhx20250054@stu.sdsmu.edu.cn (H.D.)

[2] Laboratory of Morphology, School of Basic Medical Science, Shandong Second Medical University, Weifang 261053, China; wangdongfeng@sdsmu.edu.cn

* Correspondence: liuzixuan@sdsmu.edu.cn

† These authors contributed equally to this work.

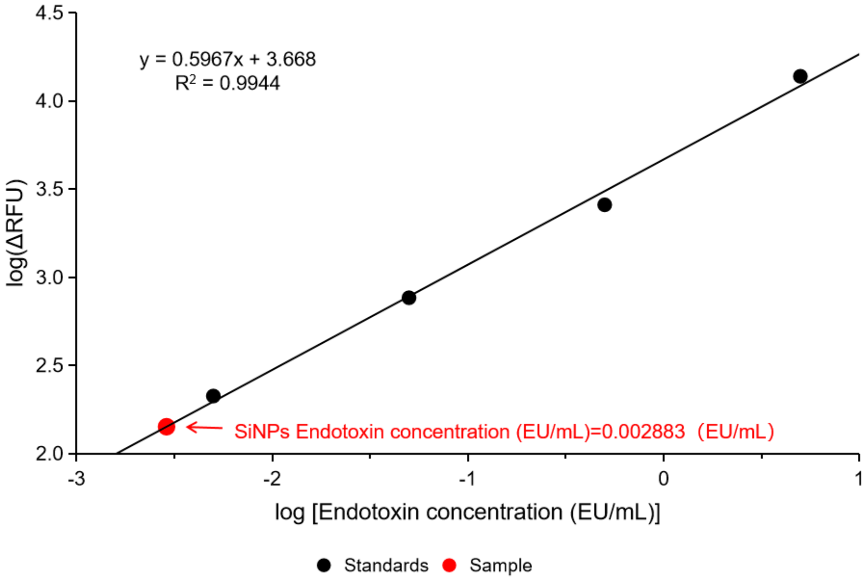

Figure S1. Endotoxin level of SiNPs. The endotoxin concentration was measured in SiNPs suspension at 1 mg/mL using an Endotoxin Detection Kit. The measured value was 0.0029 EU/mL, which is below the detection limit of the kit (0.005 EU/mL EU/mL).

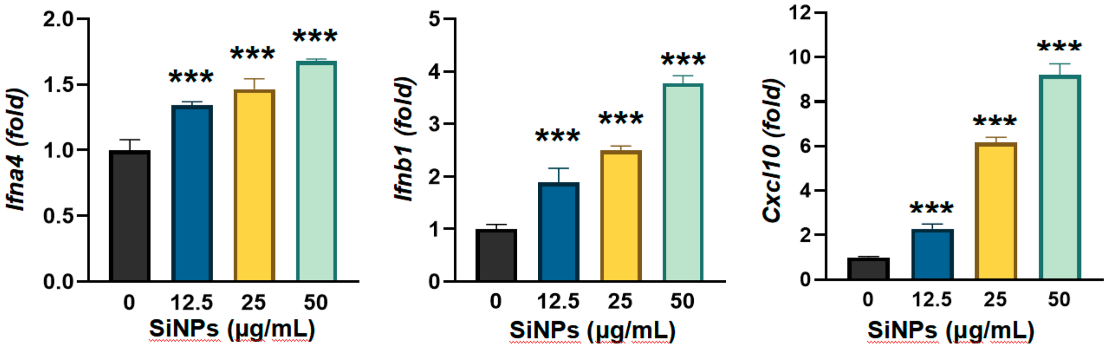

Figure S2. Ifna4, Ifnb1 and Cxcl10 transcripts measured by real-time PCR. Data from three independent experiments were expressed as the means ± SD. ***$p < 0.001$ compared to the 0 group. All data are expressed as mean ± SD.

Table S1. Summary of the physical properties of SiNPs.

	SiNPs in Water	SiNPs in Saline	SiNPs in DMEM(with 10% FBS)
Size (nm)	189.23±1.7	1206.67±148.4	1363.67±169.5
Zeta Potential (mV)	-32.1±3.0	-23.4±2.3	-13.7±3.6

Table S2. Primer Sequences

Gene	Gene Description Primer sequence (5'-3')
Il-18-F	GTT TAC AAG CAT CCA GGC ACA
Il-18-R	TCA TGC AGC CTC GGG TAT TC
Il-6-F	CCC CAA TTT CCA ATG CTC TCC
Il-6-R	CGC ACT AGG TTT GCC GAG TA
Il-1β-F	AAT GAAGGA ACG GAG CC
Il-1β-R	CTC CAG CCA AGC TTC CTT GT
Cxcl10-F	CCA AGT GCT GCC GTC ATT TTC
Cxcl10-R	GGC TCG CAG GGA TGA TTT CAA
Ifnb1-F	CAG CTC CAA GAA AGG ACG AAC
Ifnb1-R	GGC AGT GTA ACT CTT CTG CAT
Ifna4-F	CAA TGG CTA GGC TCT GTG CT
Ifna4-R	CCT TCA GGC AGG AAA GAG GG
Gapdh-F	ACT CCA CTC ACG GCA AAT TC
Gapdh-R	TCT CCT ATG GTG ACG GCA
