# Supplementary material for: Silica Nanoparticles Elicit Pulmonary Inflammation via STING-Dependent Activation of NF-κB p65 Signaling Pathway
Source: Biomolecules. 2026 Sep 3;16(9):1273. doi: 10.3390/biom16091273 (PMC13604276; doi:10.3390/biom16091273)
Supplement: Supplementary file 1 [file biomolecules-16-01273-s001.zip › biomolecules-4478988-western blots-Figure S3.pdf]

Fig2

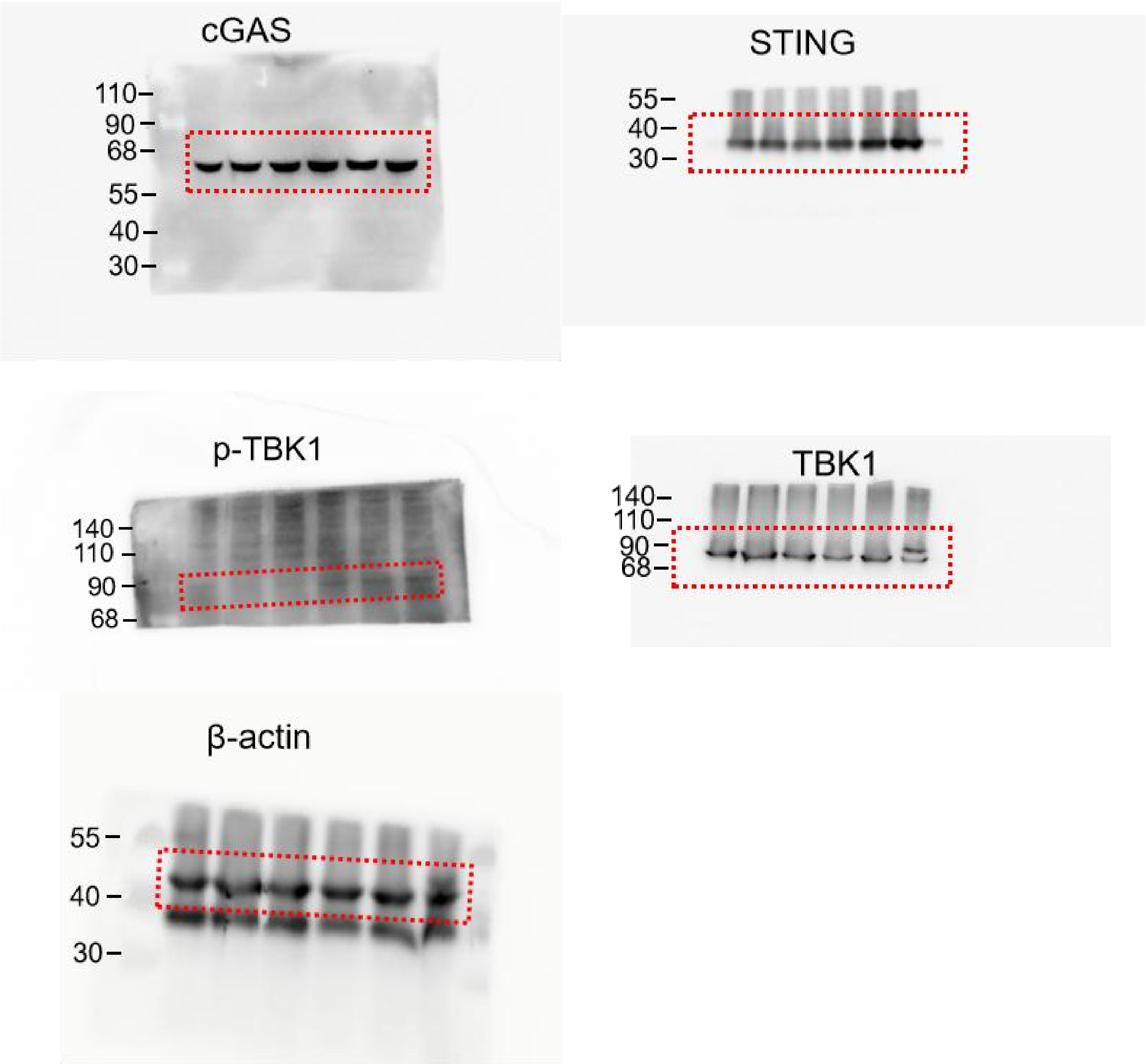
cGAS
110
90
68
55
40
30
STING
55
40
30
p-TBK1
140
110
90
68
TBK1
140
110
90
68
β-actin
55
40
30

Fig4a

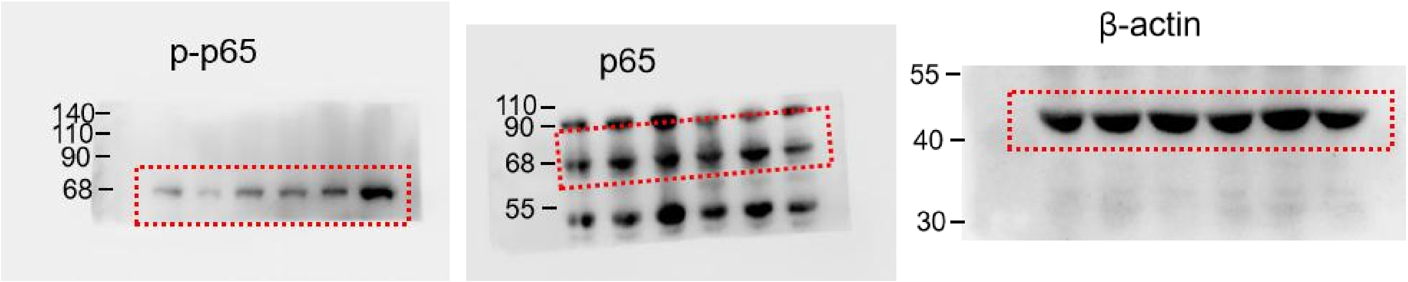

Fig4c

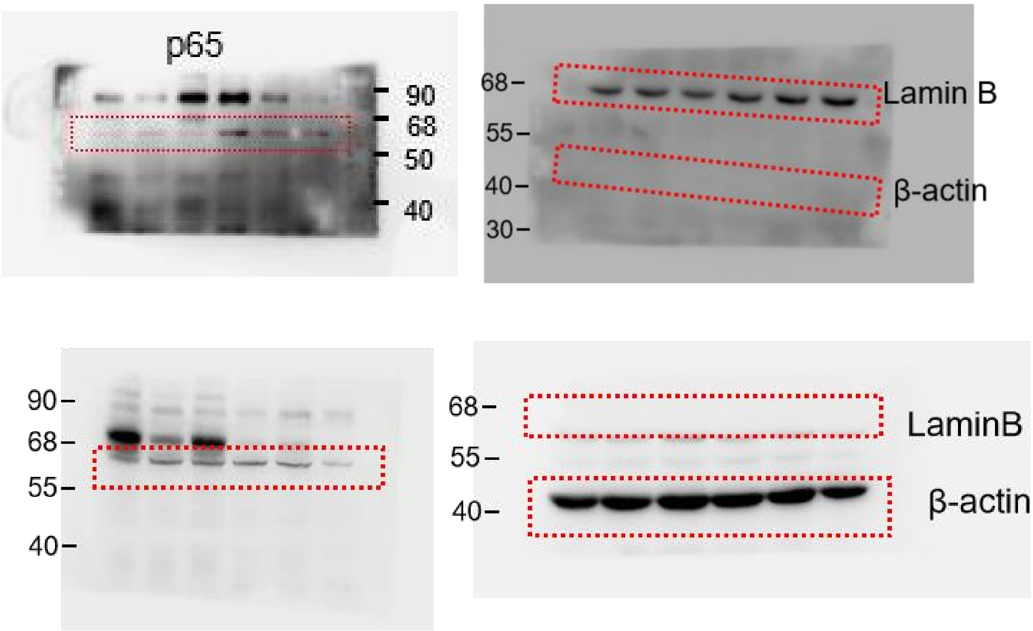

Fig4d

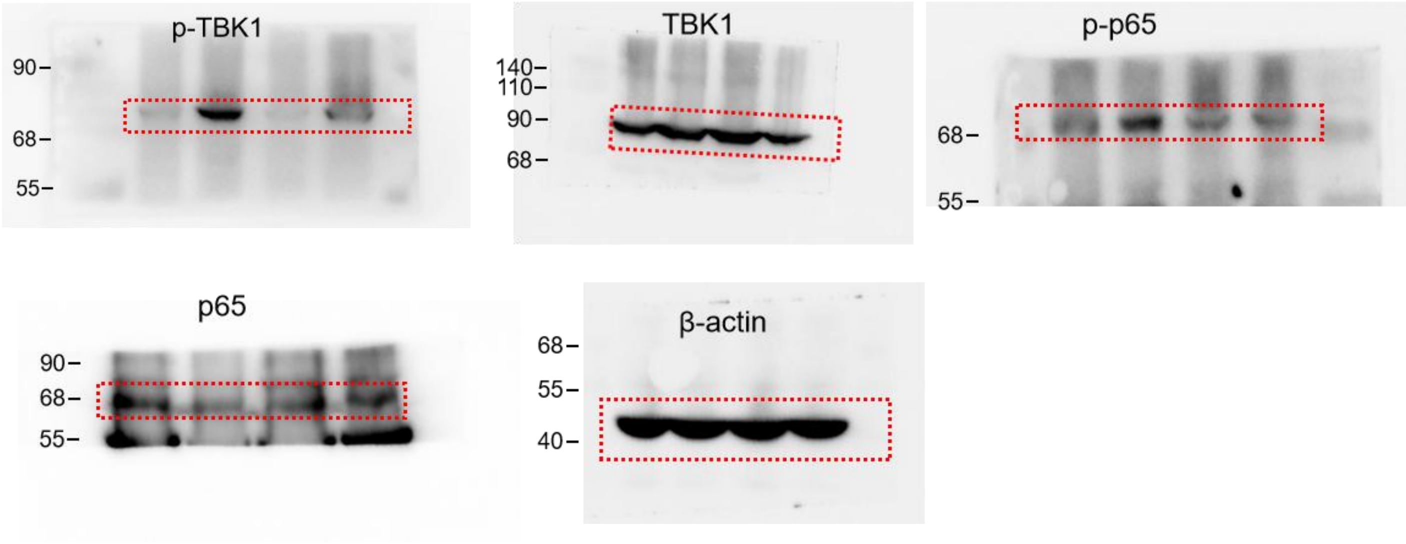

Fig6

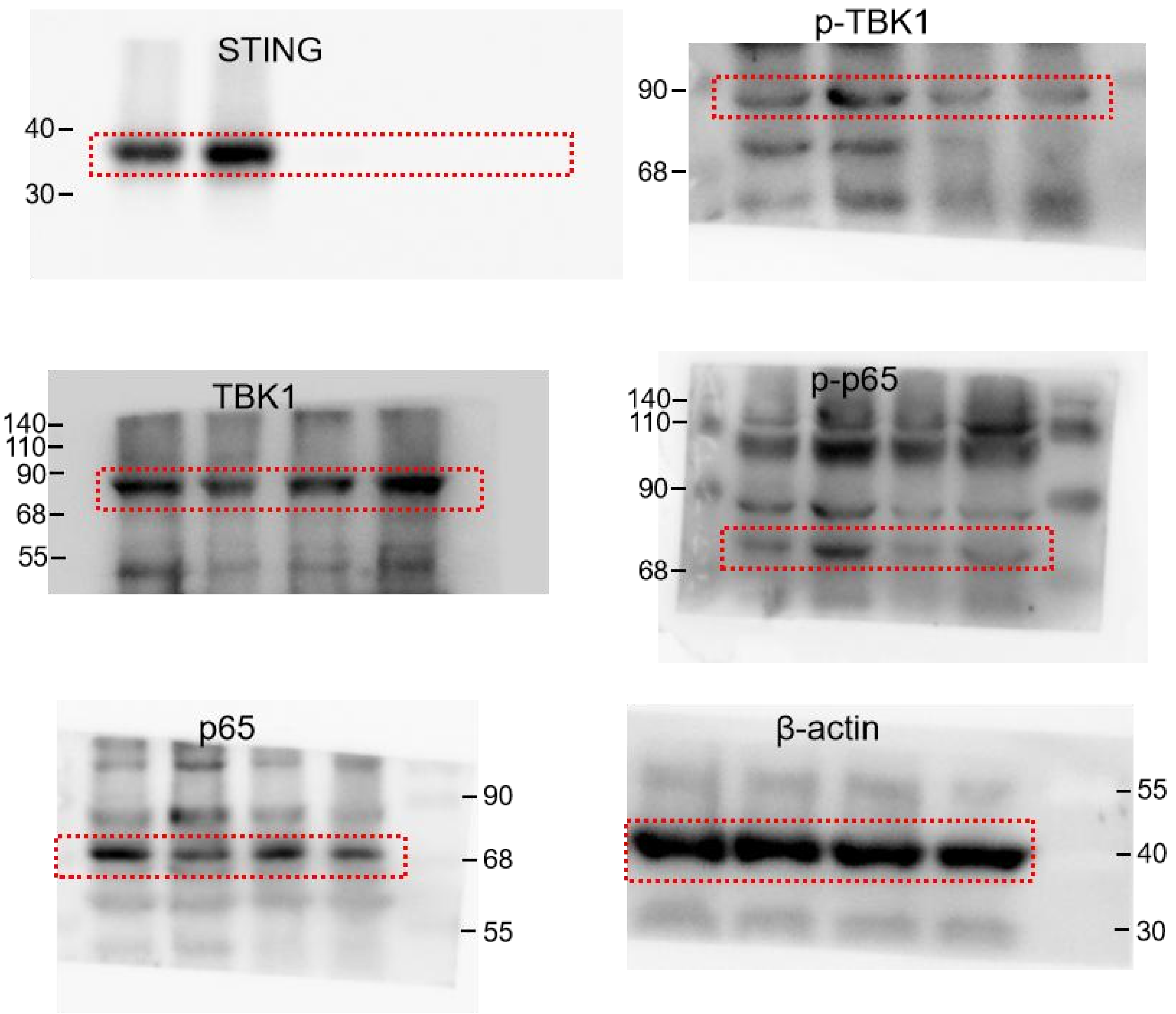
STING
40
30
p-TBK1
90
68
TBK1
140
110
90
68
55
p-p65
140
110
90
68
p65
90
68
55
β-actin
55
40
30
